# The proteasome subunit *psmb1* is essential for craniofacial cartilage maturation and morphogenesis

**DOI:** 10.1172/jci.insight.181723

**Published:** 2024-07-18

**Authors:** Bess M. Miller, Wolfram Goessling

**Affiliations:** 1 Division of Genetics, Brigham and Women’s Hospital, Harvard Medical School, Boston, Massachusetts, USA.; 2 Broad Institute of MIT and Harvard, Cambridge, Massachusetts, USA.; 3 Harvard Stem Cell Institute, Cambridge, Massachusetts, USA.; 4 Harvard-MIT Division of Health Sciences and Technology, Cambridge, Massachusetts, USA.; 5 Division of Gastroenterology, Massachusetts General Hospital, Boston, Massachusetts, USA.

**Keywords:** Development, Cartilage, Embryonic development

## Abstract

Craniofacial dysmorphisms are among the most common birth defects. Proteasome mutations frequently result in craniofacial dysmorphisms, including lower jaw malformations; however, the underlying mechanisms are unknown. Here, we used a zebrafish proteasome subunit β 1 (*psmb1*) mutant to define the cellular mechanisms underlying proteasome mutation-induced craniofacial dysmorphisms. *psmb1* mutants exhibited a flattened ceratohyal and smaller Meckel’s and palatoquadrate cartilages. Ceratohyal flattening was a result of failed chondrocyte convergent extension, accompanied by reduced numbers of chondrocytes in the lower jaw due to defects in chondrocyte differentiation. Morphogenesis of craniofacial muscles and tendons was similarly perturbed. *psmb1* mutants lacked the hyohyal muscles, and craniofacial tendons were shortened and disorganized. We additionally identified a critical period for proteasome function in craniofacial development, specifically during chondrocyte and muscle differentiation. *psmb1* overexpression in *sox10^+^* cells of mutant embryos rescued both cartilage and tendon phenotypes but induced only a partial rescue of the muscle phenotype, indicating that *psmb1* was required in both tissue-autonomous and nonautonomous fashions during craniofacial development. Overall, our work demonstrates that *psmb1* is required for craniofacial cartilage, tendon, and muscle differentiation and morphogenesis.

## Abstract 2

The proteasome is required in a time- and tissue-specific manner during craniofacial development.

## Introduction

Craniofacial development requires precisely synchronized morphogenesis of tissues derived from the ectoderm, mesoderm, and endoderm to form and integrate the muscles, tendons, cartilages, and bones of the face. Craniofacial disorders account for one-third of birth defects in the United States and affect 1 in 600 births (1, 2). Craniofacial disorders have a wide range of severity and contribute significantly to infant mortality, as they can interfere with breathing and feeding (1). Even less severe craniofacial disorders can result in serious functional and social deficits that impact quality of life. The genetic and environmental etiology of craniofacial disorders is complex, and over 700 distinct syndromes have been described (3). A common cause of craniofacial dysmorphisms is prenatal exposure to alcohol, which can result in fetal alcohol spectrum disorders (FASDs). FASDs are estimated to affect 1%–5% of school age children in the United States and Western Europe (4–8). Fetal alcohol syndrome, the most severe category of FASDs, is characterized by central nervous system defects, growth delay, and craniofacial dysmorphisms (9).

Our group has previously found that developmental exposure to ethanol inhibits the ubiquitin-proteasome system and that treatment of zebrafish embryos with proteasome inhibitors induces malformations in Meckel’s cartilage (10), indicating that loss of proteostasis may contribute to craniofacial anomalies in patients FASD. In support of this, several studies have recently identified craniofacial dysmorphisms in patients with proteasome subunit mutations (11–13). However, the mechanisms underlying the emergence of craniofacial malformations due to proteasome inhibition and mutation are unknown. Genetic models of proteasome mutation are therefore essential to comprehensively examine the role of the proteasome in craniofacial development.

Here, we used zebrafish mutants in the 20S proteasome subunit β 1 gene *psmb1* to identify the cellular mechanisms underlying lower jaw defects resulting from proteasome inhibition. We found that *psmb1* mutation induced widespread defects in craniofacial cartilage, tendon, and muscle morphogenesis. Craniofacial cartilage and muscle are less mature in *psmb1* mutants compared with wild-type or heterozygous counterparts, and *psmb1* mutants have fewer chondrocytes in Meckel’s cartilage, the ceratohyal, and the palatoquadrate cartilages. Overexpression of *psmb1* in the neural crest and chondrocytes rescued the cartilage and tendon phenotypes but induced only a partial rescue of the muscle phenotype, indicating that there are both tissue-autonomous and nonautonomous effects of *psmb1* on muscle development.

## Results

### psmb1^hi2939^ mutation disrupts proteasome assembly.

The *psmb1^hi2939^* line was generated by a retroviral insertional mutagenesis screen in which the virus inserted into the first intron (14–16). Therefore, we first assayed the effect of this mutation on *psmb1* expression and proteasome assembly. RT-PCR for the full-length *psmb1* transcript revealed that *psmb1* mutants produce wild-type transcript (Supplemental Figure 1A; supplemental material available online with this article; https://doi.org/10.1172/jci.insight.181723DS1). However, qRT-PCR for *psmb1* demonstrated that mutants express *psmb1* at approximately half the level of wild-type fish (Supplemental Figure 1B). To interrogate whether this mutation perturbs proteasome assembly, we performed native PAGE on 72-hour postfertilization (hpf) pooled wild-type and heterozygous versus mutant larvae using an antibody that recognizes the α 1, 2, 3, 5, 6, and 7 subunits of the proteasome (Supplemental Figure 1C). *psmb1* mutants had virtually no 19S or 26S proteasome but retained a small amount of 30S proteasome, demonstrating that this mutation substantially impairs proteasome assembly.

### psmb1 mutation results in fewer craniofacial chondrocytes and impaired ceratohyal elongation.

**Figure 1:**
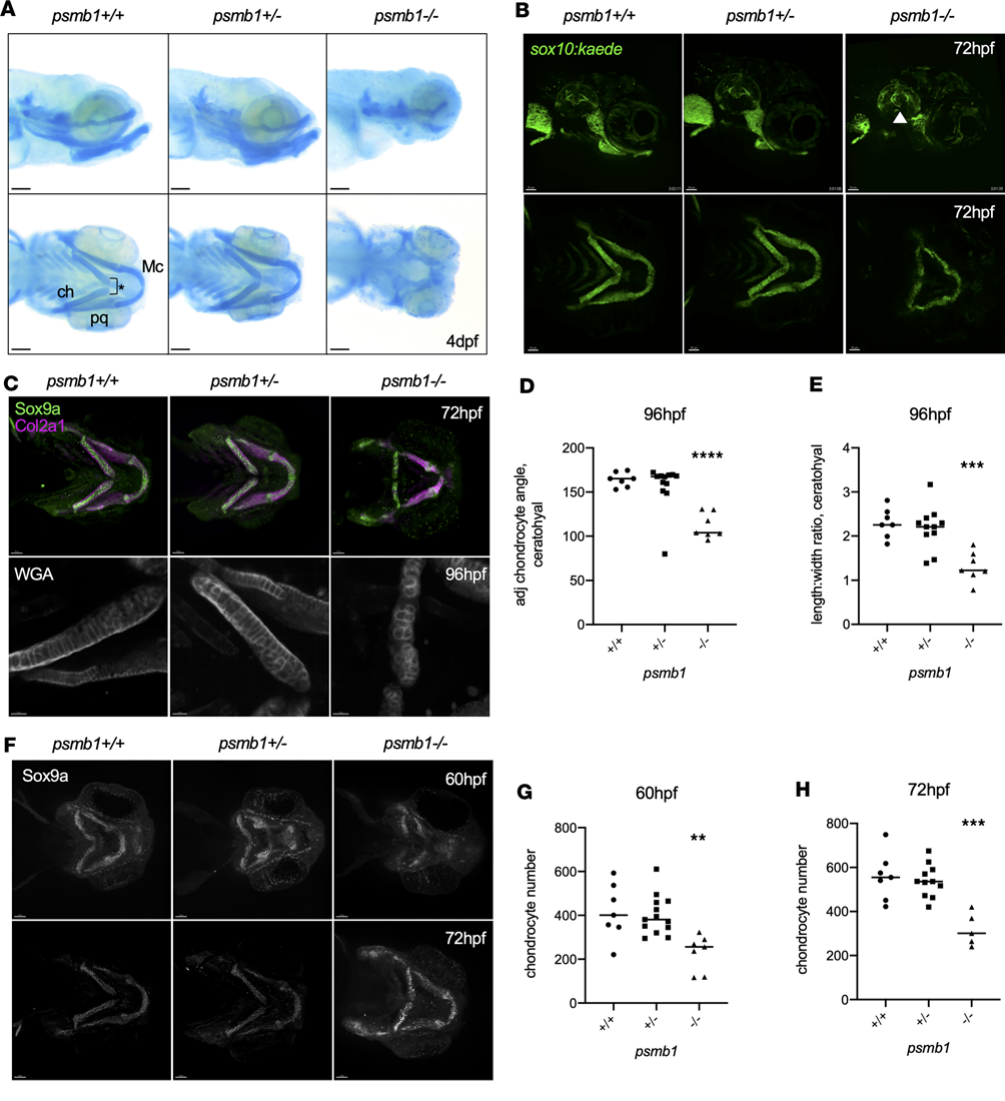
Failure of chondrocyte convergent extension in *psmb1* mutants. (**A**) Alcian blue staining of 4 dpf *psmb1* larvae. Mc, Meckel’s cartilage; ch, ceratohyal; pq, palatoquadrate. The asterisk indicates ceratohyal angle. Scale bars: 100 μm. (**B**) Imaging of *sox10:kaede; psmb1^–/–^* larvae at 72 hpf demonstrates defects in first and second arch cartilage derivatives as well as semicircular canal formation (white arrowhead). Scale bars: 50 μm. (**C**) Col2a1 (magenta) and Sox9a (green) antibody staining of *psmb1* larvae at 72 hpf (top, scale bars: 50 μm) and wheat germ agglutinin stain at 96 hpf (bottom, scale bars: 30 μm). (**D**) Quantification of chondrocyte stacking in wheat germ agglutinin stain images via analysis of the angle formed by drawing a line through 3 adjacent chondrocytes. *n* = 7 (+/+), 12 (+/–), 7 (–/–). (**E**) Quantification of chondrocyte stacking in wheat germ agglutinin stain images by analysis of length/width ratio. *n* = 7 (+/+), 12 (+/–), 7 (–/–). (**F**) Antibody stain for Sox9a at 60 hpf and 72 hpf. Scale bars: 50 μm. (**G**) Quantification of the number of chondrocytes present in the ceratohyal, palatoquadrate, and Meckel’s cartilages at 60 hpf in images in **F**. *n* = 7 (+/+), 13 (+/–), 7 (–/–). (**H**) Quantification of the number of chondrocytes present in the ceratohyal, palatoquadrate, and Meckel’s cartilages at 72 hpf in images in **F**. *n* = 7 (+/+), 11 (+/–), 5 (–/–). Data shown represent mean ± SD. Significance was calculated with 1-way ANOVA with Dunnett’s multiple comparisons test, **P ≤ 0.01, ***P ≤ 0.001, ****P ≤ 0.0001.

*psmb1* mutants have reduced or absent Meckel’s, ceratohyal, and palatoquadrate cartilages, accompanied by flattening of the ceratohyal when visible, as assessed by Alcian blue staining (10) (Figure 1A). In addition to defects in cartilage development, *psmb1* mutants also have significantly reduced expression of liver and pancreas genes; however, they do not suffer from general growth restriction, as measured by larval length (10). Additionally, heart and kidney development are both morphologically normal in *psmb1* mutants (Supplemental Figure 2), further demonstrating that the observed developmental defects are tissue-specific and not the result of a general growth delay phenotype.

As Alcian blue is a relatively late marker of cartilage formation, we imaged *sox10:kaede; psmb1^–/–^* larvae at 72 hpf to investigate the cellular basis of this phenotype (Figure 1B). *sox10* is expressed in the neural crest early in development and is strongly expressed in chondrocytes starting at 2 days postfertilization (dpf) (17). Imaging of *sox10:kaede*; *psmb1^–/–^* larvae revealed a similar phenotype to Alcian blue staining, although less severe. While Meckel’s, palatoquadrate, and ceratohyal chondrocytes were present in *psmb1* mutants, these cartilages were reduced in size (Figure 1B). The disparity in cartilage robustness between Alcian blue staining and presence of *sox10^+^* chondrocytes suggests that *psmb1* mutant chondrocytes are less able to produce extracellular matrix (ECM). Imaging of the *sox10:kaede* reporter line also revealed defects in semicircular canal formation in *psmb1* mutants (Figure 1B, white arrowhead), similar to those seen in patients with *PSMC3* mutations (12). To further investigate the chondrocyte differentiation stage, we performed antibody staining for Sox9a, a key regulator of chondrogenesis, and Col2a1, a major component of the chondrocyte ECM, both of which were produced by *psmb1^–/–^* chondrocytes (Figure 1C), indicating that chondrocytes begin on at least the early stages of differentiation.

Ceratohyal elongation during the 55–72 hpf time window is largely due to chondrocyte convergent extension (18, 19). If this process happens appropriately, chondrocytes stack next to each other such that drawing a line through the center of 3 adjacent chondrocytes yields a flat, 180-degree angle. Stacking is also associated with increased length/width ratio in chondrocytes. Given the flattened ceratohyal angle in *psmb1* mutants, we next asked whether chondrocyte cell movements were impaired. To investigate this question, we used wheat germ agglutinin staining to visualize chondrocyte cell membranes and quantify stacking (Figure 1C). *psmb1* mutant chondrocytes were disorganized compared with wild-type and heterozygous fish and had a reduced length/width ratio (Figure 1, D and E). These results suggest that chondrocyte convergent extension is defective in *psmb1* mutants, although reduced chondrocyte numbers likely also contribute to morphological defects in the ceratohyal cartilages.

To better visualize ceratohyal elongation, we next performed time-lapse imaging of *sox10:kaede; psmb1^–/–^* fish from 55 to 70 hpf (Supplemental Video 1). At 55 hpf, *psmb1^–/–^* larvae were largely indistinguishable from wild-type and heterozygous larvae, although the mutants exhibited a 1- to 2-hour developmental delay. However, elongation of the ceratohyal cartilage was completely lost in *psmb1* mutants, further supporting impaired chondrocyte convergent extension. Given the reductions in the lower jaw cartilages, we also used Sox9a antibody staining to quantify the number of chondrocytes in these cartilages throughout development (Figure 1F). By 60 hpf, *psmb1* mutants had approximately half the number of chondrocytes as wild-type and heterozygous fish, and this difference persisted through 72 hpf (Figure 1, G and H). These results demonstrate that *psmb1* is required for chondrocyte convergent extension and that 55–65 hpf represents a critical period for proteasome function during chondrocyte development.

**Figure 2:**
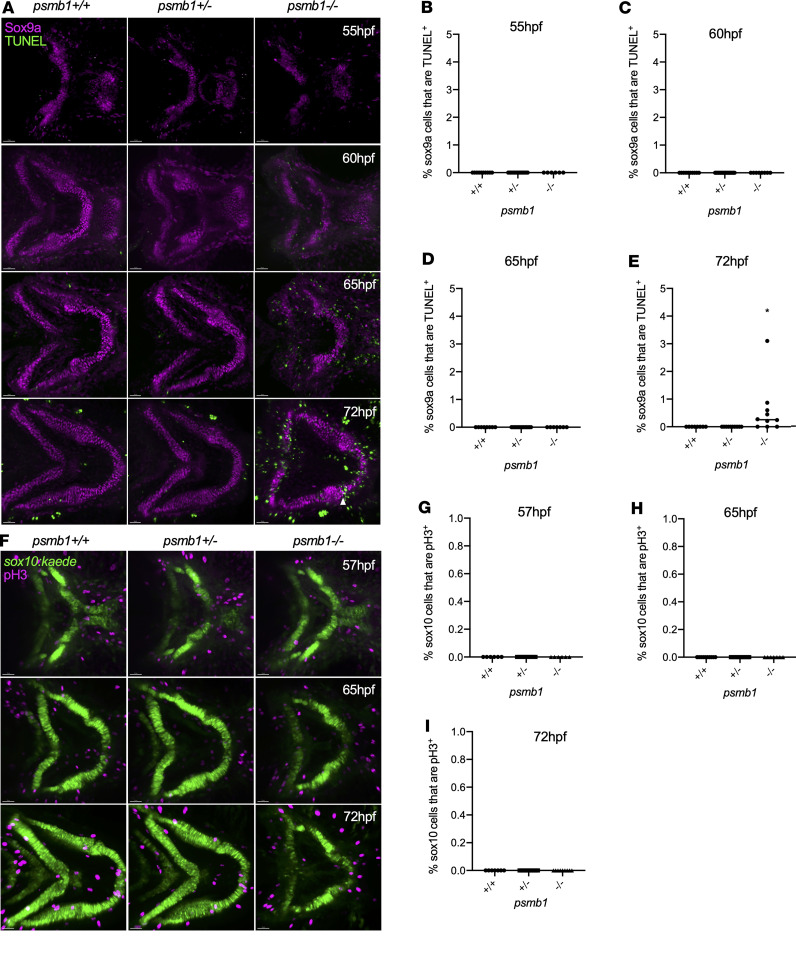
Chondrocyte cell death and proliferation in *psmb1* mutants. (**A**) Time-course study of apoptag TUNEL staining (green) and Sox9a staining (magenta) from 55–72 hpf. (**B**–**E**) Quantification of **A** demonstrates that cell death is not elevated in mutant chondrocytes until 72 hpf, at which point sox9a^+^TUNEL^+^ cells can be found (white arrowhead). 55 hpf: *n* = 10 (+/+), 16 (+/–), 6 (–/–). 60 hpf: *n* = 10 (+/+), 21 (+/–), 8 (–/–). 65 hpf: *n* = 6 (+/+), 6 (+/–), 6 (–/–). 72 hpf: *n* = 8 (+/+), 10 (+/–), 10 (–/–). (**F**) pH3 (magenta) antibody staining in *sox10:kaede* larvae (green) at 57 hpf, 65 hpf, and 72 hpf. (**G**–**I**) Quantification of **C** shows no proliferation of craniofacial chondrocytes across genotypes. 57 hpf: *n* = 6 (+/+), 10 (+/–), 6 (–/–). 65 hpf: *n* = 10 (+/+), 14 (+/–), 7 (–/–). 72 hpf: *n* = 7 (+/+), 11 (+/–), 9 (–/–). Scale bars: 50 μm. Data shown represent mean ± SD. Significance was calculated with 1-way ANOVA with Dunnett’s multiple comparisons test. *P < 0.05; ns, not significant.

As elevated cell death is seen in the head of *psmb1* mutants by 55 hpf (10), we next performed a time-course study of TUNEL staining from 55 to 72 hpf to determine whether decreases in chondrocyte number in the mutants are attributable to increased cell death. We found increased numbers of Sox9a^+^TUNEL^+^ cells in mutants at 72 hpf but not at earlier stages (Figure 2, A–E). Interestingly, we also observed increased numbers of Sox9a^–^TUNEL^+^ perichondral cells lining the outer edge of the mutant cartilages at 65 hpf and 72 hpf, and cell death was also globally elevated in noncartilage tissues of the head at 65 and 72 hpf (Figure 2A). We additionally employed antibody staining for phospho-histone H3 (pH3) to evaluate chondrocyte proliferation at 57–72 hpf in *sox10:kaede; psmb1^–/–^* embryos. No pH3^+^sox10^+^ cells were present at any time point examined in either mutant or wild-type/heterozygous larvae (Figure 2, F–I), which agrees with results of previous studies of chondrocyte proliferation during this time window (18). Therefore, neither increases in chondrocyte cell death nor decreases in proliferation drive the observed reductions in chondrocyte numbers.

### Pharyngeal arch morphogenesis and patterning occurs normally in psmb1 mutants.

**Figure 3:**
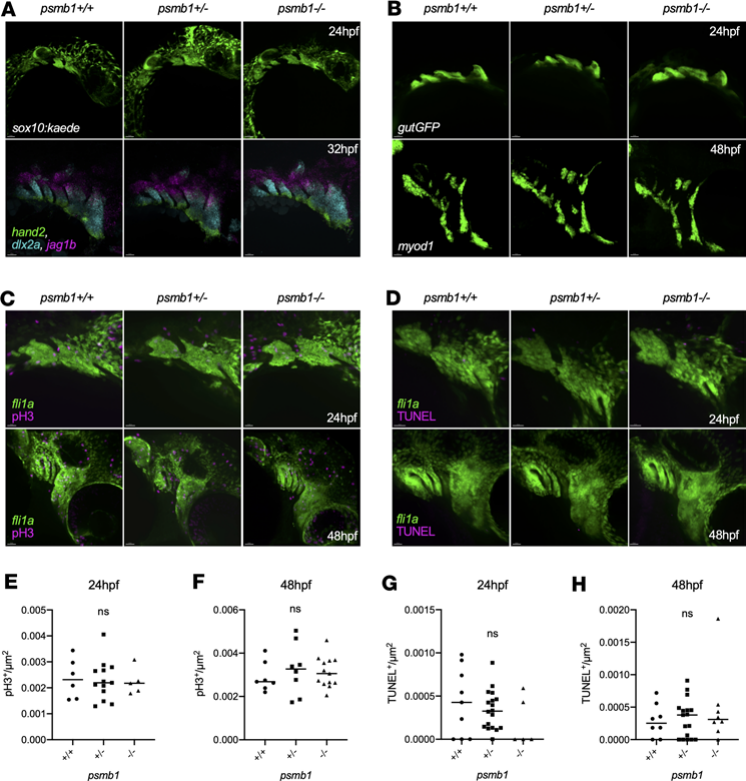
*psmb1* is dispensable for pharyngeal arch morphogenesis and patterning. (**A**) Imaging of *sox10:kaede* embryos at 24 hpf (top, scale bars: 50 μm) did not reveal any defects with neural crest cell migration to the arches. *RNAscope* for *hand2*, *dlx2a*, and *jag1b* demonstrates normal dorsal-ventral arch patterning in *psmb1* mutants at 32 hpf (bottom, scale bars: 30 μm). (**B**) Imaging of *gutGFP* embryos at 24 hpf and *myod1* RNAscope at 48 hpf demonstrates that the pharyngeal arch endoderm and muscle are formed normally in *psmb1* mutants. Scale bars: 50 μm. (**C**) pH3 antibody staining (magenta) in *fli1a:EGFP* embryos at 24 hpf and 48 hpf to assess neural crest cell proliferation in *psmb1* mutants. Scale bars: 30 μm (**D**) TUNEL staining (magenta) in *fli1a:EGFP* embryos at 24 hpf and 48 hpf to assess neural crest cell death in *psmb1* mutants. Scale bars: 20 μm. (**E** and **F**) Quantification of proliferation in pharyngeal arches 1 and 2 in images in **C**; normalization is to arch 1 and 2 *fli1a^+^* area. (**E**): *n* = 6 (+/+), 13 (+/–), 5 (–/–). (**F**): *n* = 7 (+/+), 8 (+/–), 13 (–/–). (**G** and **H**) Quantification of cell death in pharyngeal arches 1 and 2 in images in **D**; normalization is to *fli1a^+^* arch 1 and 2 area. (**G**): *n* = 9 (+/+), 18 (+/–), 5 (–/–). (**H**): *n* = 8 (+/+), 17 (+/–), 8 (–/–). Data shown represent mean ± SD. Significance was calculated with 1-way ANOVA with Dunnett’s multiple comparisons test, ns: not significant.

Meckel’s cartilage, the ceratohyal, and the palatoquadrates are derived from the first and second pharyngeal arches; therefore, we next asked whether defects in these cartilages could be traced back to issues with pharyngeal arch development. Imaging of *sox10:kaede; psmb1^–/–^* embryos at 24 hpf did not reveal any defects in neural crest cell (NCC) patterning in the arches (Figure 3A). RNAscope for *hand2*, *jag1b*, and *dlx2a* at 32 hpf to assess dorsal-ventral patterning within the pharyngeal arches was also normal in *psmb1* mutants (Figure 3A). Finally, we used the *gutGFP* reporter line and RNAscope for *myod1* to visualize the development of the pharyngeal endoderm and muscle, respectively, both of which were morphologically normal in *psmb1* mutants (Figure 3B). As such, the mutant craniofacial phenotype is not caused by defects in either NCC migration or patterning within the arches or issues with pharyngeal endoderm or mesoderm development.

Next, we asked whether changes in NCC proliferation or cell death could be contributing to reductions in chondrocyte number. We quantified TUNEL^+^*fli1a*^+^ and pH3^+^*fli1a*^+^ cells in the first and second pharyngeal arches at 24 and 48 hpf to specifically assay the cells that give rise to Meckel’s cartilage, the palatoquadrates, and the ceratohyal cartilages. No differences in proliferation or cell death of pharyngeal arch NCCs (Figure 3, C–H) or in pharyngeal arch area at 24 hpf or 48 hpf (Supplemental Figure 3) were observed. Combined with our finding that *psmb1^–/–^* larvae have fewer craniofacial chondrocytes, this indicates that there is likely reduced differentiation of pharyngeal arch NCCs into chondrocytes.

### Proteasome subunit genes are upregulated in a tissue-specific fashion in psmb1 mutants.

**Figure 4:**
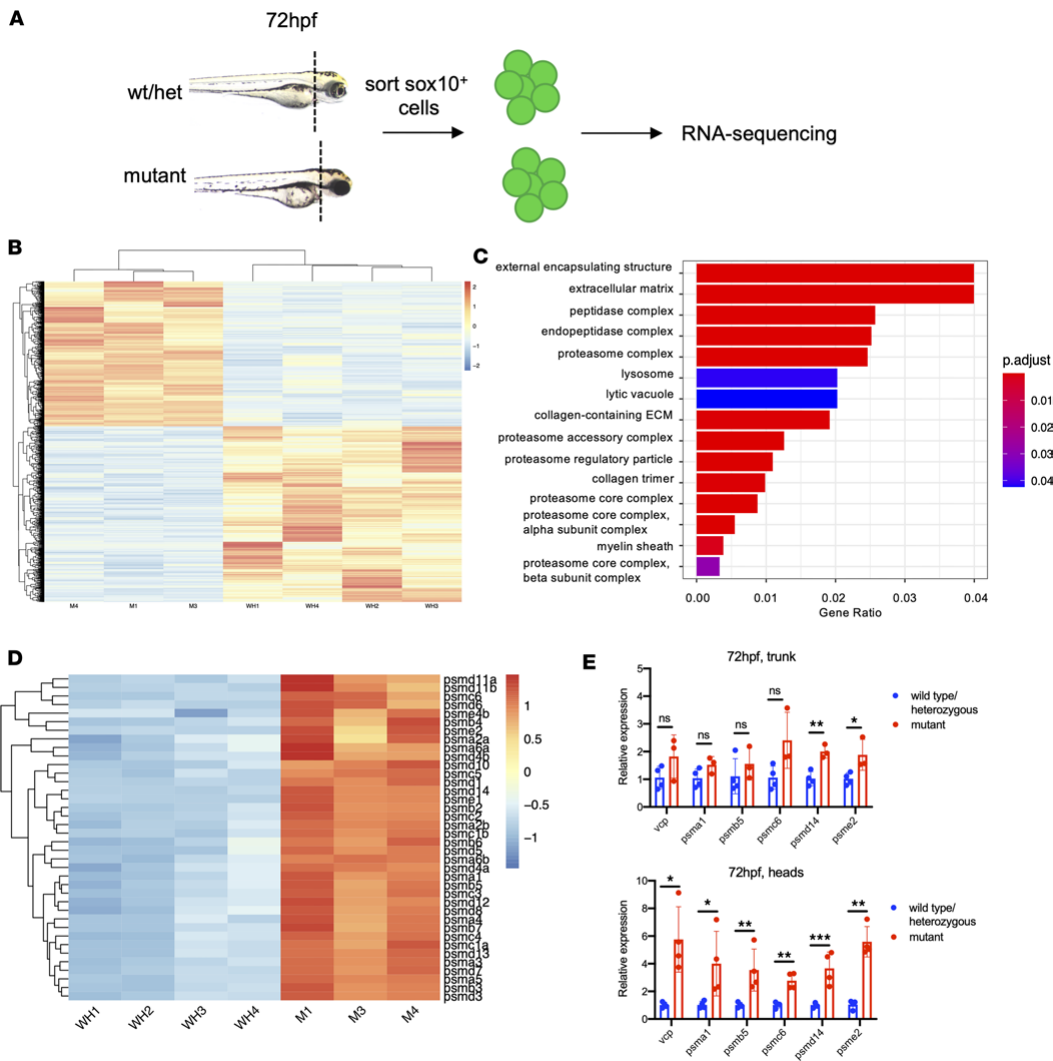
Proteasome subunit genes are upregulated in a tissue-specific fashion in *psmb1* mutants. (**A**) Schematic of RNA-Seq approach for cranial *sox10*^+^ cells from wild-type/heterozygous versus mutant fish at 72 hpf. (**B**) Heatmap of normalized counts of differentially expressed genes in cranial *sox10*^+^ cells at 72 hpf. Wald’s test with false discovery rate correction (*P* adjusted < 0.05). Each sample represents cells from 25 different larvae. (**C**) GO enrichment analysis using ClusterProfiler identifies GO components enriched in the differentially expressed gene set. (**D**) Heatmap of proteasome subunit gene expression in RNA-Seq data set from sorted cranial *sox10^+^* cells (**E**) Top: qRT-PCR for proteasome subunit genes on cDNA from dissected trunks/tails at 72 hpf. Bottom: qRT-PCR for proteasome subunit genes on cDNA from dissected heads at 72 hpf. WH, pooled wild-type/heterozygous larvae; M, mutant larvae. Data shown represent mean ± SD. Significance was calculated with unpaired *t* test. *P < 0.05, **P < 0.01, ***P < 0.001, ****P < 0.0001.

The molecular mechanisms linking proteasome mutations and craniofacial defects are unknown; therefore, we next performed RNA-Seq of craniofacial chondrocytes to assess transcriptional alterations that result from *psmb1* mutation. To do so, we dissected heads from 72-hpf mutants and pooled wild-type and heterozygous larvae and sorted *sox10^+^* cells (Figure 4A). As the *Tg(sox10:kaede)zf393* line is expressed in non-CNCC cell types, such as the ear, we were unable to exclude all non-CNCC contamination; however, this reporter is expressed most strongly in chondrocytes at 72 hpf. There were 2,308 differentially expressed genes in this data set, and Gene Ontology (GO) analysis revealed alterations primarily in pathways related to the proteasome and ECM composition (Figure 4, B and C). As is typically seen following proteasome inhibition (20), proteasome subunit genes were strongly upregulated in *psmb1* mutants (Figure 4D). qRT-PCR of trunk/tail tissue versus dissected heads alone at 72 hpf revealed that upregulation of proteasome subunit genes occurred in a tissue-specific manner, with tissues in the head demonstrating much stronger upregulation of proteasome subunit genes (Figure 4E). These results suggest that cranial tissues are more sensitive to *psmb1* mutation than tissues in the trunk, which may explain the relative severity of craniofacial phenotypes in *psmb1* mutants.

We also observed widespread downregulation of genes involved in chondrocyte maturation and ECM production, which we verified via qRT-PCR of dissected heads (Supplemental Figure 4). As we sorted equal numbers of cells from *psmb1* mutant and wild-type/heterozygous fish, reduced ECM gene expression could reflect either reduced numbers of maturing chondrocytes in the sox10^+^ population or reduction in ECM gene expression in individual chondrocytes. We did not see decreased expression of *sox9a* or *barx1* (Supplemental Figure 4A), suggesting the sorted populations contain roughly equivalent numbers of chondrocytes. Thus, these results demonstrate that proteasome function is required for craniofacial chondrocyte maturation in addition to morphogenesis.

### Muscle and tendon defects in psmb1 mutants.

**Figure 5:**
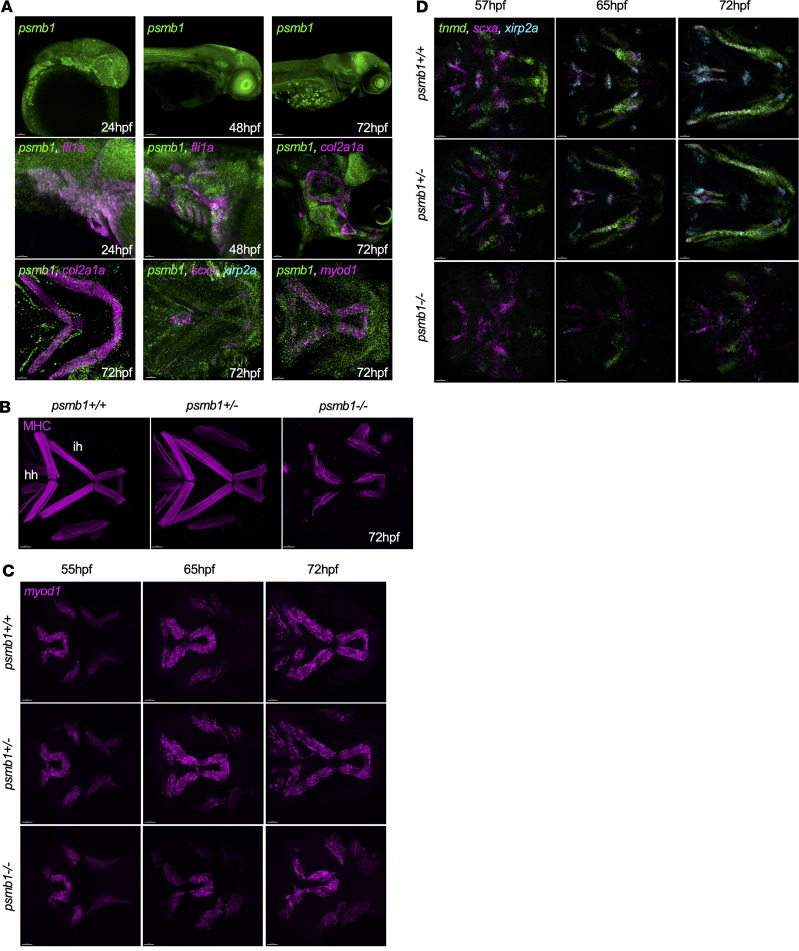
Craniofacial muscle and tendon defects in *psmb1* mutants. (**A**) RNAscope to assess developmental timing and distribution of *psmb1* expression. Top: *psmb1* (green) expression at 24, 48, and 72 hpf. Scale bars: 50 μm, 100 μm, and 100 μm, respectively. Middle: *psmb1* expression in developing pharyngeal arches and cartilage. Left and middle: *psmb1* (green), *fli1a* (magenta), right: *psmb1* (green), *col2a1a* (magenta). Scale bars: 50 μm. Bottom: *psmb1* expression in craniofacial tissues at 72 hpf. Left: *psmb1* (green), *col2a1a* (magenta). Middle: *psmb1* (green), *myod1* (magenta). Right: *psmb1* (green), *scxa* (magenta), *xirp2a* (cyan). Scale bars: 30 μm. (**B**) Antibody stain for myosin heavy chain (MHC) at 72 hpf showing defects in craniofacial muscles in *psmb1* mutants. Scale bars: 50 μm. hh, hyohyal; ih,interhyal. (**C**) RNAscope for *myod1* at 55–72 hpf. Scale bars: 30 μm. (**D**) RNAscope for *tnmd* (green), *scxa* (magenta), and *xirp2a* (cyan) at 57–72 hpf demonstrating defects in tendon development in *psmb1* mutants. Scale bars: 30 μm.

Using RNAscope, we found that *psmb1* was expressed throughout the embryo during development, with particularly high expression in the brain, pharyngeal arches, and eye (Figure 5A). We additionally determined that *psmb1* is expressed ubiquitously throughout craniofacial development, including in the pharyngeal arches at 24 and 48 hpf and in craniofacial chondrocytes, muscle, and tendons at 72 hpf (Figure 5A). This observation prompted us to examine the development of craniofacial muscles and tendons in *psmb1* mutants. Antibody staining for myosin heavy chain (MHC) at 72 hpf showed that *psmb1* mutants lack hyohyal muscles and have reduced interhyal, intermandibularis anterior, and intermandibularis posterior muscles (Figure 5B). Myoblast fusion may also be impaired in *psmb1* mutants, as the cells are more distinct and rounded than in wild-type and heterozygous larvae. Time-lapse imaging of *myf5:EGFP*; *mylz2:mCherry*; *psmb1^–/–^* fish from 55 to 70 hpf revealed that muscles that form later in development, specifically the hyohyal, were more severely affected (Supplemental Video 2). As MHC is a fairly late marker of muscle formation, we additionally performed a RNAscope time-course study of *myod1* expression, which validated loss of the hyohyal muscles (Figure 5C). Both time-lapse imaging and *myod1* expression confirmed that while *psmb1* mutants looked similar to wild-type and heterozygous fish at 55 hpf, by 65 hpf they had sharply diverged. This timing is concordant with the appearance of defects in craniofacial cartilage and strongly suggests that proteasome function is specifically required for proper craniofacial development between 55 and 65 hpf.

RNA-Seq of cranial *mylz2^+^* cells at 72 hpf, comparing dissected heads from mutant larvae with those from pooled wild-type and heterozygous larvae, revealed 5,106 differentially expressed genes in total. GO analysis indicated alterations in muscle differentiation and development terms as well as proteasome-related terms (Supplemental Figure 5, A and B). We found overall downregulation of muscle differentiation genes, including the transcription factors *myod1* and *myf5*, genes involved in myoblast fusion such as *myomaker*, *jam2a*, and *jam3b* (21–24), and multiple myosin and actin genes (Supplemental Figure 5C), confirmed by qRT-PCR on dissected heads (Supplemental Figure 5D). These results indicate that *psmb1* mutation hinders muscle differentiation.

To examine tendon development, we additionally performed RNAscope at 57–72 hpf for the tendon markers *scxa* and *tnmd1* and for *xirp2a*, which marks myosepta and sites of muscle attachment (25). This revealed defects in tendon formation, including disorganization of the mandibulohyoid and hyohyal junctions and smaller palatoquadrate tendons (Figure 5D). These defects were apparent as early as 57 hpf. Furthermore, time-lapse imaging of *scxa:mCherry; col2a1a:EGFP; psmb1^–/–^* embryos from 55 to 70 hpf (Supplemental Video 3) demonstrated that although tendon cells are initially specified, their ability to coalesce into tendons is substantially diminished in *psmb1* mutants, altogether demonstrating that *psmb1* is required for tendon maturation and morphogenesis.

### Overexpression of psmb1 in the neural crest and chondrocytes rescues cartilage and tendon defects in psmb1 mutants.

Cranial NCCs regulate muscle patterning and differentiation during craniofacial development (26–28), and defects in muscle differentiation and function can likewise result in cartilage phenotypes (29, 30). This raises the question of whether defects in craniofacial muscle and cartilage development in *psmb1* mutants are a result of tissue-autonomous loss of *psmb1* in individual tissues or indirect nontissue autonomous loss of *psmb1* across tissues.

**Figure 6:**
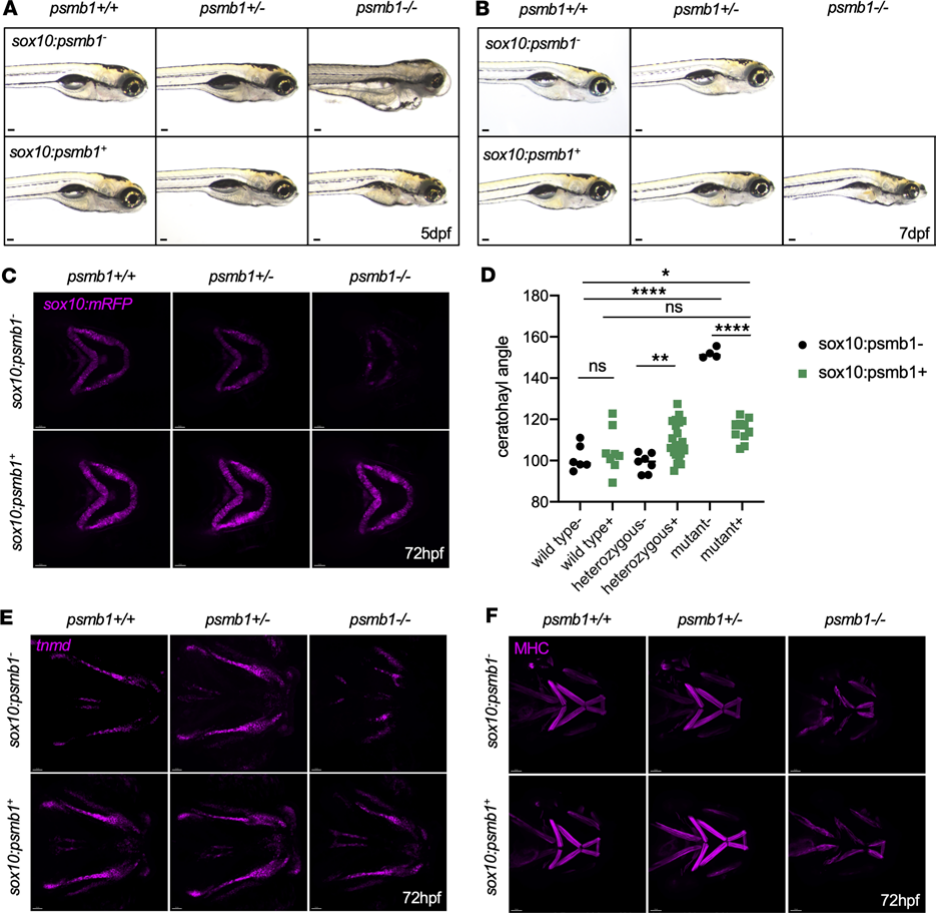
Overexpression of *psmb1* in the neural crest and chondrocytes rescues mutant cartilage and tendon phenotype. (**A**) Bright-field images of *psmb1* wild-type, heterozygous, and mutant larvae with and without *sox10:psmb1-2A-GFP* transgene at 120 hpf. Scale bars: 100 μm. (**B**) Bright-field images of *psmb1* wild-type, heterozygous, and mutant larvae with and without *sox10:psmb1-2A-GFP* transgene at 7 dpf. Scale bars: 100 μm. (**C**) Confocal imaging of *sox10:mRFP, psmb1* wild-type, heterozygous, and mutant larvae with and without *sox10:psmb1-2A-GFP* transgene at 72 hpf. Scale bars: 50 μm. (**D**) Quantification of ceratohyal angle in images from **C** demonstrates that ceratohyal angle is almost completely restored in rescued *psmb1* mutants. Data shown represent mean ± SD. *n* = 6, 8, 7, 22, 4, 10. Significance was calculated with 2-way ANOVA. *P < 0.05, **P < 0.01, ****P < 0.0001; ns, not significant. (**E**) *sox10:psmb1-2A-GFP* transgene substantially improves tendon phenotype, as assessed by RNAscope for *tnmd* at 72 hpf. Scale bars: 50 μm. (**F**) *sox10:psmb1-2A-GFP* partially rescues muscle phenotype, as assessed by antibody staining for myosin heavy chain (MHC) at 72 hpf. Scale bars: 50 μm.

To disentangle tissue autonomous and nonautonomous effects of *psmb1* mutation, we generated a *sox10:psmb1-2A-GFP* transgenic line, in which *psmb1* was overexpressed in the neural crest and cartilage. *psmb1^–/–^* larvae died at 5 dpf, at which point there was widespread tissue death and edema (10) (Figure 6A). Overexpression of *psmb1* in *sox10^+^* cells reversed edema and tissue duskiness at 5 dpf and extended survival to at least 7 dpf, although rescued mutants still failed to inflate the swim bladder and demonstrated abnormal head shape compared with wild-type/heterozygous siblings (Figure 6, A and B). This experiment demonstrates that edema is primarily a function of the mutation’s effect on *sox10*^+^ cells and their descendants.

Overexpression of *psmb1* under the *sox10* promoter was also sufficient to rescue ceratohyal patterning in *psmb1* mutants (Figure 6, C and D), indicating that tissue-specific loss of *psmb1* in the neural crest and chondrocytes drives defects in cartilage morphogenesis. Tendons in *sox10:psmb1-2A-GFP; psmb1^–/–^* larvae were also elongated, better organized, and more robust compared with their unrescued counterparts (Figure 6E). This rescue is likely a primary effect of *psmb1* overexpression in *sox10^+^* NCCs from which tendons are derived, but improvements in ceratohyal patterning may also contribute to this improvement, as craniofacial cartilage is required for tendon cell organization (25). Finally, *sox10:psmb1-2A-GFP; psmb1^–/–^* larvae displayed a modest partial rescue of the muscle as well. Notably, hyohyal muscles were completely lacking in control *psmb1^–/–^* larvae, but rudimentary, shortened hyohyal muscles were visible in *sox10:psmb1-2A-GFP; psmb1^–/–^* larvae (Figure 6F). These results indicate that *psmb1* is required in a tissue-autonomous manner during cartilage and tendon development and in both tissue-autonomous and nontissue autonomous fashions during muscle development.

## Discussion

Proteasome subunit mutations in *PSMB1*, *PSMC3*, and *PSMD12* are associated with craniofacial dysmorphisms in humans, including microcephaly, semicircular canal malformations, and retrognathia and microretrognathia, respectively (11–13). Patients with these mutations also display neurodevelopmental phenotypes such as intellectual disability and developmental delay (13). Our group and others have found that pharmacologic or genetic proteasome inhibition causes abnormalities in the shape of Meckel’s cartilage in zebrafish (10, 11, 13). However, both whether proteasome subunit mutations have more comprehensive effects on craniofacial development and the cellular mechanisms connecting proteasome inhibition with defects in craniofacial cartilage are unknown. Here, we found that *psmb1* mutation induced widespread developmental defects in first and second arch–derived craniofacial tissues. Additionally, we identified a critical period for proteasome function during craniofacial cartilage and muscle morphogenesis and maturation starting at 55–60 hpf. As *psmb1* is maternally deposited in zebrafish (13), a lack of earlier phenotypes may be due to either maternal compensation or reduced requirement for *psmb1* at earlier stages of development. Notably, proteasome subunit expression is more strongly upregulated in the head than in the rest of the body in *psmb1* mutants, indicating that cranial tissues are more highly sensitized to proteasome dysfunction than tissues in the trunk. This may explain the predominance and severity of craniofacial phenotypes in these mutants.

In addition to impairing chondrocyte maturation, our data indicate that *psmb1* mutation also decreases upstream differentiation of NCCs into chondrocytes. Although *psmb1* mutants had fewer craniofacial chondrocytes than wild-type or heterozygous fish by 60 hpf, we did not observe differences in chondrocyte cell death or proliferation prior to 72 hpf in *psmb1* mutants, indicating that reduced chondrocyte numbers were due to impaired NCC differentiation into chondrocytes. Reduced NCC numbers could also explain this phenotype; however, we did not find differences in pharyngeal arch NCC area in *psmb1* mutants. The discrepancy between reduced chondrocyte numbers and a seemingly normal NCC population raised the question of what happens to NCCs that fail to differentiate into chondrocytes. Given that we saw increased TUNEL^+^Sox9a^–^ perichondral cells at 72 hpf, one possibility is that these cells do indeed go on to die at later time points, losing their lineage marker in the process.

Proteasome inhibition has previously been found to influence differentiation, proliferation, and apoptosis of chondrocytes in long bone growth plates. Treatment of young mice with proteasome inhibitors has been found to decrease long bone length (31–33). Accompanying in vitro studies of fetal rat metatarsal bones found that proteasome inhibitors reduced chondrocyte proliferation and hypertrophy and increased chondrocyte apoptosis, which was accompanied by upregulation of p53 (31–33). Inhibition of apoptosis rescued long bone growth in vivo, demonstrating that the proteasome inhibition-induced chondrocyte apoptosis directly contributes to defects in long bone growth (34). This is in contrast to our finding that chondrocyte apoptosis does not drive craniofacial phenotypes, which may be due to differences in craniofacial versus long bone chondrogenesis or due to differences in developmental stage.

Proteasome inhibition has not previously been reported to induce defects in craniofacial muscle development. The proteasome, however, is known to be a regulator of skeletal muscle differentiation. Proteasome inhibition prevents fusion in primary myoblast cultures and results in dysregulation of myogenic gene expression (35–37). Mutations in *PSMB8* result in muscle atrophy in humans, and fast-twitch muscle-specific knockout of *PSMC3* results in muscle growth defects and a decrease in force production in mice (38, 39). Muscle growth defects in this model are accompanied by histological changes indicative of muscle degeneration, including decreased myofiber size, central nuclei, and accumulation of basophilic inclusions (38). In line with these studies, we saw dysregulation of myogenic gene expression in *psmb1* mutants as well as disorganization of myofibrils, demonstrating that the role of the proteasome in muscle differentiation extends to the craniofacial muscle, despite its distinct embryonic origin and differences in the upstream network governing myogenic regulatory factor expression and activity. In contrast to the muscle and cartilage, very little is known concerning the role of the proteasome in tendon development and maintenance. Our results indicate that the proteasome is dispensable for tendon specification but plays an important role in tendon morphogenesis.

In sum, our study identifies a critical period for proteasome function during craniofacial development, from 55 to 65 hpf. Prior to this period, craniofacial development proceeds broadly normally in *psmb1* mutants; however, starting at 55 hpf alterations in craniofacial cartilage, muscle, and tendon morphogenesis and differentiation are apparent. These findings demonstrate that there are defined tissue- and temporally specific roles for the proteasome during craniofacial development.

## Methods

### Sex as a biological variable.

Experiments were performed on clutch-matched siblings that were used without sex bias, as it is not possible to distinguish the sex of larval zebrafish. Each experiment was performed at least twice to validate results across clutches.

### Zebrafish studies.

If not specified, fish used in this study were wild-type strain AB. Lines used in this study included *Tg(sox10:kaede)zf393* (40), *Tg(scxa:mCherry)* (41), *Tg(myf5:EGFP)* (42), *Tg(mylz2:mCherry)* (43)*,* *Tg(gutGFP)* (44), *Tg(fli1a:EGFP)* (45), *psmb1*^hi2939^ (14, 16, 46), and *Tg(sox10:psmb1-2A-GFP)*. We thank the lab of Leonard Zon (Boston Children’s Hospital, Boston, Massachusetts, USA) for sharing the *Tg(sox10:kaede)* line; the lab of Jenna Galloway (Massachusetts General Hospital) for sharing the *Tg(myf5:EGFP)*, *Tg(mylz2:mCherry)*, and *Tg(scxa:mCherry)* lines; and the lab of Trista North (Boston Children’s Hospital) for sharing the *Tg(fli1a:EGFP)* line. *psmb1^hi2939^* embryos were obtained from ZIRC and genotyped upon retrieval to confirm identity. Embryos were raised in E3 at 28.5C.

### Genotyping.

DNA was extracted from tail clips or whole larvae by incubating tissue in 50 mM NaOH for 30 minutes at 95°C. NaOH was neutralized with 1 M Tris pH 8, and DNA was then used for genotyping. Two PCRs were used to genotype *psmb1* fish — one to detect the wild-type allele and one to detect the transgenic insertion. Primer sequences are provided in Supplemental Table 1.

### Transgenesis.

The transgenic line *Tg(sox10:psmb1-2A-GFP)* was generated using Tol2 transgenesis. Full-length *psmb1* was cloned from AB cDNA using primers specified in Supplemental Table 1. The middle entry vector pENTR:psmb1 was generated using the pENTR/D-TOPO kit (Thermo Fisher Scientific, K59120). The p5E:sox10 plasmid was a gift from the Galloway lab. Gateway reactions using LR Clonase II Plus (Invitrogen 12538120) were performed to generate the pTol2 *sox10:psmb1-2A-GFP; cryaa:cerulean* plasmid. *Tol2* mRNA was synthesized using the mMessage mMachine SP6 in vitro transcription kit (Ambion, AM1340) according to the manufacturer’s instructions. To generate stable lines, single-cell embryos from a *psmb1^+/–^* in cross were injected with 1–2 nL of 25 ng/μL plasmid and 25 ng/μL *Tol2* mRNA. F0s were screened for GFP expression, raised to adulthood, and crossed to AB fish to identify founders. F1 fish were then raised to adulthood and crossed to *psmb1^+/–^* fish to generate embryos for experiments.

### RNAscope.

RNAscope was performed using the RNAscope Multiplex Fluorescent V2 Assay (Advanced Cell Diagnostics, 323100). Methods were modified from those of refs. 47, 48. Briefly, embryos were fixed overnight at 4°C in 4% PFA and then dehydrated through a methanol series. Embryos in methanol were stored at –20°C for at least 2 hours. Embryos were rehydrated, washed with PBS 0.1% Tween, and deyolked. Embryos were permeabilized with proteinase K 20 μg/mL according to age (0 min for 0–1.5 dpf, 1 min for 2 dpf, and 3 min for 3 dpf). Embryos were post fixed with 4% PFA for 20 minutes, washed with PBS 0.1% Tween, and incubated with probe diluted according to the manufacturer’s specifications overnight at 40°C. Following probe incubation all remaining protocol steps were performed according to ref. 47. The following probes were used: *psmb1* (ACD, 1185011-C1), *col2a1a* (ACD, 409471-C3), *myod1* (ACD, 481231-C2), *scxa* (ACD, 564451-C2), *xirp2a* (ACD, 564471-C3), *tnmd* (ACD, 564481-C1), *dlx2a* (ACD, 52352-C3), *jag1b* (ACD, 1189981-C2), *hand2* (ACD, 500051-C1), *egfp* (ACD, 400281-C2), and *gfp* (ACD, 1192271-C2). Background subtraction and deconvolution were run on each image using Imaris 10.0.0.

### Immunofluorescence.

Embryos were fixed in 4% PFA overnight at 4°C and then washed 4 times with PBS 0.1% Tween. Embryos were permeabilized with proteinase K 20 μg/mL according to age (2.5 min for 48 hpf and 5 min for 72 hpf) and then post-fixed with 4% PFA for 20 minutes. Embryos were incubated in PBS 0.5% Triton X-100 for 2 hours and then blocked in PBS with 5% normal goat serum, 5% BSA for 1 hour. Embryos were incubated in primary antibody overnight in blocking solution at 4°C and then washed 6 times with PBS 0.1% Tween. Embryos were then incubated in secondary antibodies in block overnight at 4°C, washed 6 times with PBS 0.1% Tween, and then embedded in 0.6% agarose for imaging. The following primary antibodies were used: Sox9a (GeneTex, GTX128370, 1:100), Collagen Type II (DSHB, II-II6B3, 1:50), MHC (DSHB, A4.1025, 1:50), and GFP (Aves, GFP-1020, 1:100). The following secondary antibodies were used at 1:250 dilution as appropriate: goat anti-chicken Alexa Fluor 488 (Jackson, 703-545-155), goat anti-rabbit Alexa Fluor 568 (Invitrogen, A11011), goat anti-mouse IgG2a 647 (Invitrogen, A21241), and goat anti-mouse IgG1 568 (Invitrogen, A21124).

### Chondrocyte measurements.

96 hpf larvae were stained with wheat germ agglutinin Alexa Fluor 647 (Thermo Scientific, W3246, 1:500) as described above. ImageJ (NIH) was used to quantify stacking and chondrocyte length/width ratio. Stacking was measured by drawing a line through the center of 3 adjacent chondrocytes. All measurements were taken from chondrocytes in the midsections of the ceratohyal. For each larva, multiple chondrocytes were measured, and measurements were averaged for the graph. Chondrocyte counting was performed using the spots function in Imaris on Sox9a antibody-stained larvae.

### Apoptag.

TUNEL reactions on fish older than 48 hpf were carried out using the ApopTag Fluorescein In Situ Apoptosis Detection Kit (EMD Millipore, S7110). Larvae were fixed overnight in 4% PFA at 4°C, then dehydrated via a methanol series, and stored in methanol at –20°C. Larvae were rehydrated, washed with PBS 0.1% Tween, and permeabilized with 20 μg/mL proteinase K for 2–5 minutes depending on age. Larvae were postfixed for 20 minutes with 4% PFA, washed with PBS 0.1% Tween, and then incubated in 2:1 ethanol/acetic acid at –20°C for 15 minutes. Larvae were washed with PBS 0.1% Tween, incubated in equilibration buffer for 1 hour, and then incubated in TdT working solution with anti-Sox9a (GeneTex GTX128370, 1:100) overnight at 37°C. Larvae were then washed with stop/wash buffer, blocked for 1 hour with the blocking solution, and incubated in fluorescein antibody working solution with 1:250 secondary antibody goat anti-rabbit Alexa Fluor 568 (Invitrogen, A11011) overnight at 4°C. Chondrocytes were quantified using the spots function in Imaris. Only chondrocytes in Meckel’s cartilage, the palatoquadrate, and ceratohyal were included in analysis.

### TUNEL.

TUNEL reactions on fish 48 hpf and younger were carried out using the TMR Red In Situ Cell Death Detection Kit (Sigma-Aldrich, 12156792910). Embryos were fixed with 4% PFA overnight at 4°C, washed with PBS 0.1% Tween, and incubated in 2:1 ethanol/acetic acid at –20°C for 10 minutes before incubation in the TdT Enzyme/Label solution (Sigma-Aldrich, 12156792910) overnight at 37°C.

### Confocal imaging.

Larvae were embedded in 0.6% agarose and mounted on glass-bottom dishes (MatTek). Imaging was performed on an inverted Ti2 (Nikon) microscope with a Yokogawa CSuW1 spinning disk confocal and a Zyla 4.2+ sCMOS camera (ANDOR). For time-lapse imaging of developing craniofacial cartilage, muscle, and tendons, images were taken every 1 hour.

### qRT-PCR.

Depending on experiment, RNA was extracted from whole larvae, dissected trunks/tails, or dissected heads, using 10–25 larvae per group in each case. RNA was prepared using Trizol (Ambion, 15596018)/Chloroform (Sigma-Aldrich, C2432, 500 mL) isolation. 1 μg RNA per reaction was used to make cDNA with the iScript cDNA Synthesis Kit (Bio-Rad, 1708891). qRT-PCR reactions were performed using the iScript RT Supermix (Bio-Rad, 1708841). Reactions were normalized to *ef1a,* and relative expression levels calculated using the ΔΔCt method. Primer sequences are provided in Supplemental Table 1.

### RT-PCR.

RNA was extracted from 72 hpf whole larvae using Trizol/chloroform isolation. Wild-type and heterozygous embryos were pooled for RNA extraction. 1 μg RNA per reaction was used to make cDNA with the iScript cDNA Synthesis Kit (Bio-Rad, 1708891). Full-length *psmb1* transcript was amplified with the “psmb1 full-length” primers specified in Supplemental Table 1, with an expected product size of 715 bp.

### RNA-Seq.

For RNA-Seq experiments, 20–25 heads per sample were dissected from 72 hpf larvae. Dissociation was carried out using 25 μg/mL liberase (Sigma-Aldrich, 05401119001), and samples were incubated in an Eppendorf Thermomixer R at 37°C with 600 rpm shaking for 30 minutes. To encourage dissociation, samples were pipetted 20–25 times every 8–10 minutes. Samples were then passed through a 40 μm filter. Samples were sorted into Buffer RLT with 1% β-mercaptoethanol using a BD Aria Sorter with 85 μm nozzle. For RNA-Seq on *sox10:kaede^+^* cells, 7,000 cells were sorted per sample. For RNA-Seq on *mylz2^+^* cells, 1,500 cells were sorted per sample. RNA was isolated using the RNeasy MicroKit (Qiagen, 74004) and submitted to Azenta Life Sciences for library preparation and sequencing using their Ultra Low Input RNA-Seq service with 20–30 million reads per sample. The SMART-Seq HT kit was used for full-length cDNA synthesis and amplification (Takara), and the Illumina Nextera XT kit was used for sequencing library preparation. Reads were aligned to the GRCz11 reference assembly using STAR (49), and differential gene expression analysis was performed with DESeq2 (50). GO enrichment analysis was performed using clusterProfiler 4.2.2 (51).

### Native PAGE.

35 larvae per sample were lysed in buffer containing 50 mM HEPES pH 7.8, 10 mM NaCl, 1.5 mM MgCl_2_, 1 mM EDTA, 1 mM EGTA, 250 mM sucrose, and 5 mM DTT. Protein was quantified with the Pierce BSA Protein Assay Kit (Thermo Fisher Scientific, 23225). Native PAGE was performed as described in ref. 52. In lieu of a loading control, membranes were stained with 0.1% Ponceau S (Sigma-Aldrich, P3504) in 1% acetic acid following transfer. Membranes were stained with a primary antibody against proteasome 20S α subunits 1, 2, 3, 5, 6, and 7 (Enzo Life Sciences, BML-PW8195-0100, 1:5000). Secondary antibody was goat anti-mouse HRP (CST 7076S, 1:5,000).

### Statistics.

For qRT-PCR experiments, significance was calculated via unpaired 2-tailed *t* test. For comparisons across *psmb1* genotypes, significance was calculated via 1-way ANOVA with Dunnett’s multiple comparisons test. For *sox10:psmb1-2A-GFP* rescue experiments, 2-way ANOVA with multiple comparisons was utilized. *P* values of less than 0.05 were considered significant.

### Study approval.

All animal studies were approved by the Institutional Animal Care and Use Committees at the Beth Israel Deaconess Medical Center (Boston, Massachusetts, USA) and the Brigham and Women’s Hospital.

### Data availability.

RNA-Seq data sets generated for this publication have been deposited in NCBI’s Gene Expression Omnibus and are accessible through GEO accession GSE243072. Values for all data points in graphs are reported in the Supporting Data Values file.

## Author contributions

BMM contributed to conceptualization, investigation, methodology, visualization, formal analysis, writing of the original draft of the manuscript, and review and editing of the manuscript. WG contributed to conceptualization, methodology, and review and editing of the manuscript as well as resources and supervision.

## Supplementary Material

Supplemental data

Unedited blot and gel images

Supplemental video 1

Supplemental video 2

Supplemental video 3

Supporting data values

## Version 1 08/22/2024

Electronic publication
