# Supplementary material for: The proteasome subunit psmb1 is essential for craniofacial cartilage maturation and morphogenesis
Source: JCI Insight. 2024 Jul 18;9(16):e181723. doi: 10.1172/jci.insight.181723 (PMC11343588; doi:10.1172/jci.insight.181723)
Supplement: Supplemental data [file jciinsight-9-181723-s137.pdf]

Supplementary Information

Movie 1: Time-lapse imaging of *sox10:kaede*; *psmb1* embryos from 55-70hpf.

Movie 2: Time-lapse imaging of *myf5:eGFP*; *mylz2:mCherry*; *psmb1* embryos from 55-70hpf. *myf5:egfp* (green), *mylz2:mCherry* (magenta).

Movie 3: Time-lapse imaging of *col2a1a:eGFP*; *scxa:mCherry; psmb1* embryos from 55-70hpf. *col2a1a:eGFP* (green, chondrocytes), *scxa:mCherry* (orange, tendons).

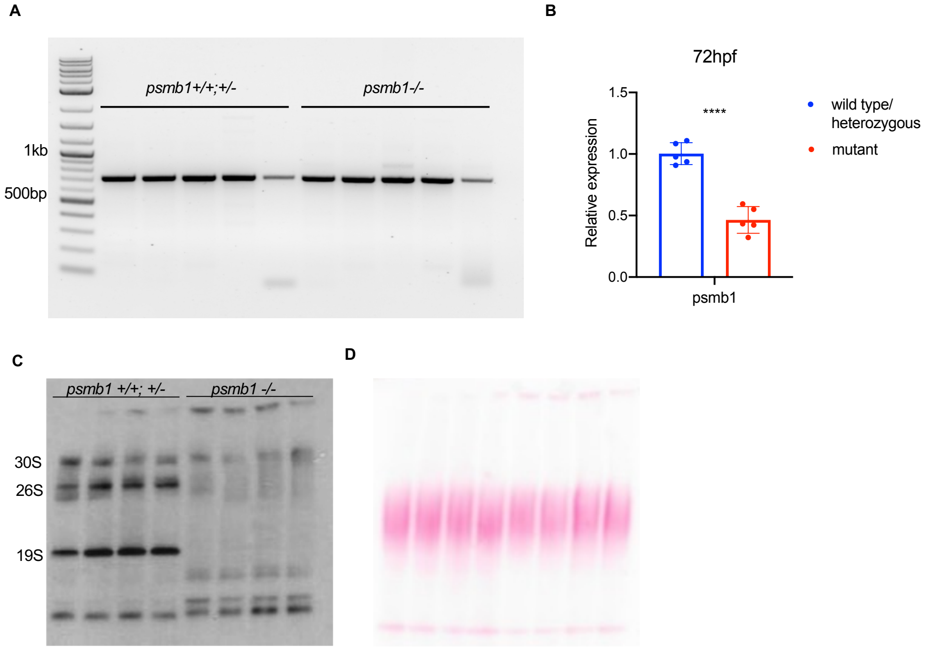

Supplementary Figure 1: *psmb1*^{hi2939} mutation perturbs proteasome assembly. A. RT-PCR for full length *psmb1* transcript in mutant larvae vs. pooled wild-type/heterozygous larvae demonstrates that *psmb1*$^{hi2939/hi2939}$ embryos make full length *psmb1* transcript. Each lane is a biological replicate. **B.** qRT-PCR for *psmb1* at 72hpf shows that *psmb1* mutants express *psmb1* at a lower level than wild-type/heterozygous fish. Unpaired t-test, error bars are mean ± SD. ****p≤0.0001. **C.** Native PAGE of 72hpf extracts from pooled wild-type/heterozygous fish vs. mutant fish probed with an antibody for proteasome 20S subunits alpha 1, 2, 3, 5, 6, 7 demonstrates defects in proteasome assembly in *psmb1* mutants. **D.** Ponceau S loading control for native PAGE in **C.**

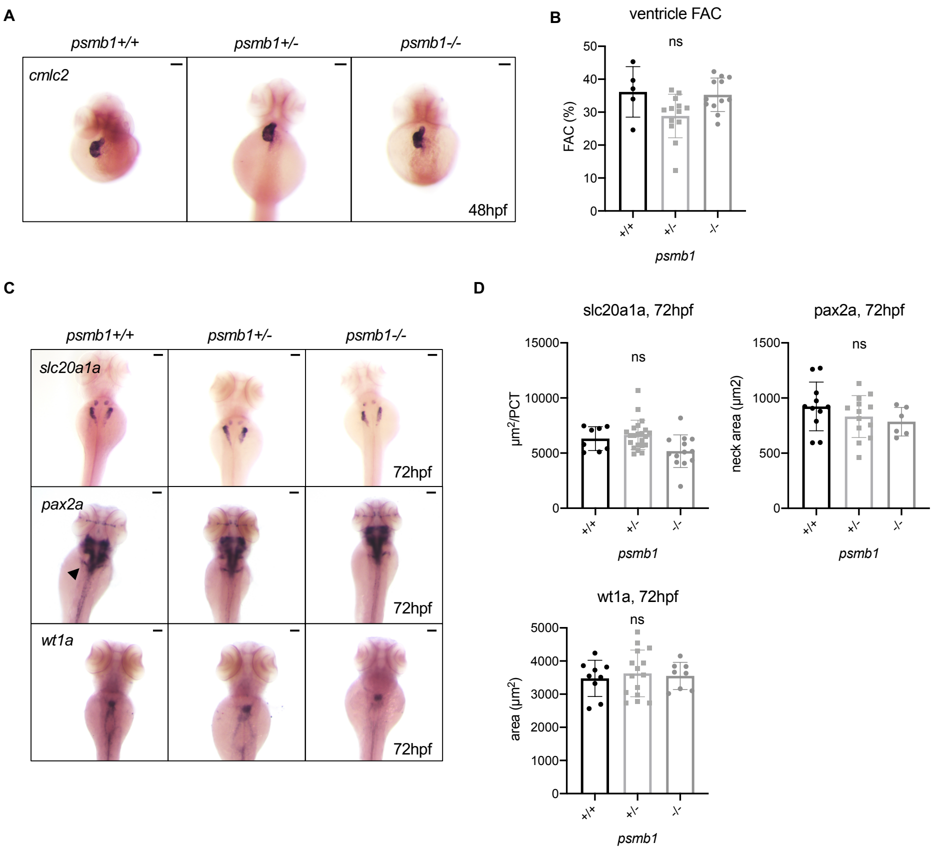

Supplementary Figure 2. *psmb1* is not required for heart or kidney development. A. ISH for *cmlc2* (cardiac muscle) at 72hpf demonstrates heart looping occurs normally. **B.** Ventricle fractional area change in *psmb1* mutants vs. wildtype and heterozygous fish, measured from brightfield movies of beating hearts. n=5, 13, 12. **C.** ISH for kidney markers *slc20a1a* (tubule, n=8, 22, 13), *pax2a* (neck, black arrowhead, n=11, 13, 6), and *wt1a* (glomerulus, n=9, 15, 8). **D.** Quantification of expression area in **C.** Data are mean + SD. ns: not significant. Scale bars are 100 µm. PCT: proximal convoluted tubule.

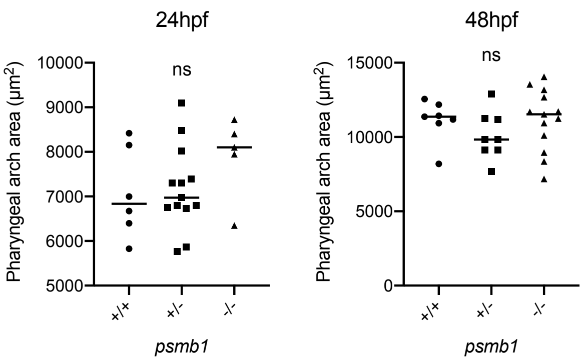

Supplementary Figure 3: Area of pharyngeal arches 1 and 2 at 24hpf (left) and 48hpf (right). Area of pharyngeal arches 1 and 2 is the same in wild-type/heterozygous and mutant fish at both 24hpf and 48hpf. 24hpf: n=10, 20, 4. 48hpf: n=7, 8, 13. Quantification is of images in **Fig. 4C** and **4D**. ns: not significant. 1-way ANOVA with Dunnett's multiple comparison test. Error bars are mean ± SD.

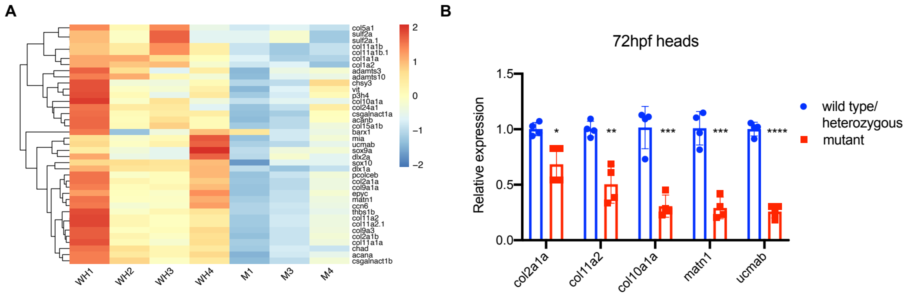

Supplementary Figure 4: RNA-sequencing demonstrates downregulation of chondrocyte maturation genes in psmb1 mutants. A. Heatmap of genes involved in chondrocyte differentiation and maturation. **B.** qRT-PCR of extracellular matrix-related gene expression on cDNA of dissected heads from *psmb1* mutant vs. wild-type/heterozygous larvae at 72hpf. Unpaired t-test, error bars are mean ± SD. WH: pooled wild-type/heterozygous larvae, M: mutant larvae. *p<0.05, **p<0.01, ***p<0.001, ****p<0.0001.

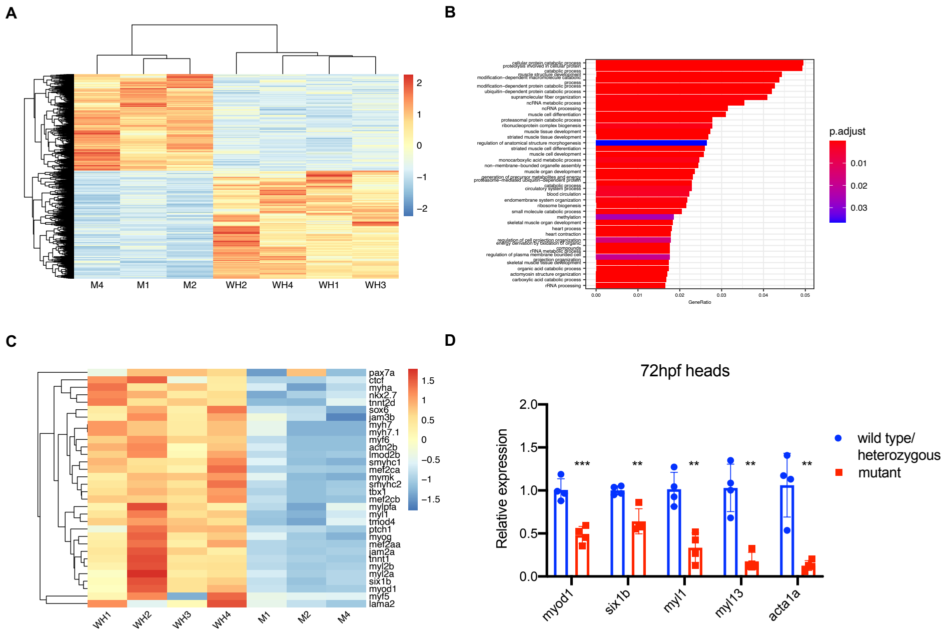

Supplementary Figure 5: RNA-sequencing of craniofacial muscle demonstrates downregulation of muscle differentiation genes in *psmb1* mutants. A. Heatmap of normalized counts of differentially expressed genes in cranial *mylz2*$^{+}$ cells at 72hpf. Wald test with false discovery rate correction (padj < 0.05). Each sample represents cells from 25 different larvae. **B.** GO enrichment analysis using ClusterProfiler identifies GO components enriched in the differentially expressed gene set. **C.** Heatmap of genes involved in muscle differentiation and maturation, which are downregulated in *psmb1* mutants. **D.** qRT-PCR of muscle differentiation genes using cDNA from dissected heads at 72hpf. Unpaired t-test, error bars are mean ± SD. WH: wild type/heterozygous, M: mutant. **p<0.01, ***p<0.001.

Gene	Forward primer	Reverse primer
psmb1 qPCR	CGGTGATACAGCGAGTTTTG	GGAGAATTTGTGCTCCACTG
psmb1 full length	CACCATGATTTCTGCCCAGGCTTATG	TCAGTCTTTCCTGAGCGGC
col2a1a qPCR	GGTCATCTGTGAGGATCTGAAT	TGGTTCTCCTTTCTGTCCCTTT
col10a1a qPCR	TGGCTCATACCACAAGGATATT	TCTCTGAGGTCAAATCGGCA
col11a2 qPCR	TCAATGGGGCTCTAGCAAAA	GGGTTTGGTTTGACTTGTTCT
matn1 qPCR	CACCCGGATCTTTCAAGTGC	TCGAAGTTCTCGGGTCTCAC
ucmab qPCR	CAGCAGCCTTTTCTTCACTC	GGCTCCATCGAATAAGGTGA
six1b qPCR	GAGGCGAAAGAAAGGGAAAAC	GAGCTCGACATCAGGGACTT
myl1 qPCR	TGTCAACTATGAGGCTTTCGT	AAATGCTTCAGTCTCCTCACA
myhz1.1 qPCR	GTGGGAGTGTGGTCAGAAGT	ATAACAAGCGGTTTTGGCAT
myod1 qPCR	CGTTCTGGAACATTACAGTGG	TGTCATAGCTGTTCCGTCTT
acta1a qPCR	CTACAGCGATGACGTAGGG	CCACCAAAATACCAAGCCAT
myl13 qPCR	CACCTTTGAGGACTTCGTTG	AAGTAGCAAGGACGTGTCTG
psmb1 wt genotyping	CAGGCTTATGGAGAAAACGGC	CGTCCCACAATAAAACAACG
psmb1 LTR genotyping	CAGGCTTATGGAGAAAACGGC	GCAGTTGCATCCGACTTGTG
vcp qPCR	ACGAGACCATTGACGCAGAG	TCTGGCTAAGAGCCCACCTA
psma1 qPCR	GCGTCAGGAGTGTTTGGACT	TGAGTCTTGCTGCCGATGAG
psmb5 qPCR	ACAAAAGAGGGCCAGGACTC	TCAAGTCGTATCGAAGGCCG
psmc6 qPCR	CAGAGTGTGGGGCAGATTGT	CCACTCTAGTGCCAGGCTTC
psmd14 qPCR	AGCGTCAAGGGAAAGGTTGT	GGTTCATGACCCAGCACCAT
psme2 qPCR	TAAACGTCTGCGTCTCTCTGC	TGGCGGTAGTTTTCTATCCTCAC
ef1a qPCR	GCGTCATCAAGAGCGTTGAG	TTGGAACGGTGTGATTGAGG

Supplementary Table 1: Primer sequences
