# Supplementary material for: The proteasome subunit psmb1 is essential for craniofacial cartilage maturation and morphogenesis
Source: JCI Insight. 2024 Jul 18;9(16):e181723. doi: 10.1172/jci.insight.181723 (PMC11343588; doi:10.1172/jci.insight.181723)

Fig. S1A – RT-PCR for psmb1, all lanes included in figure

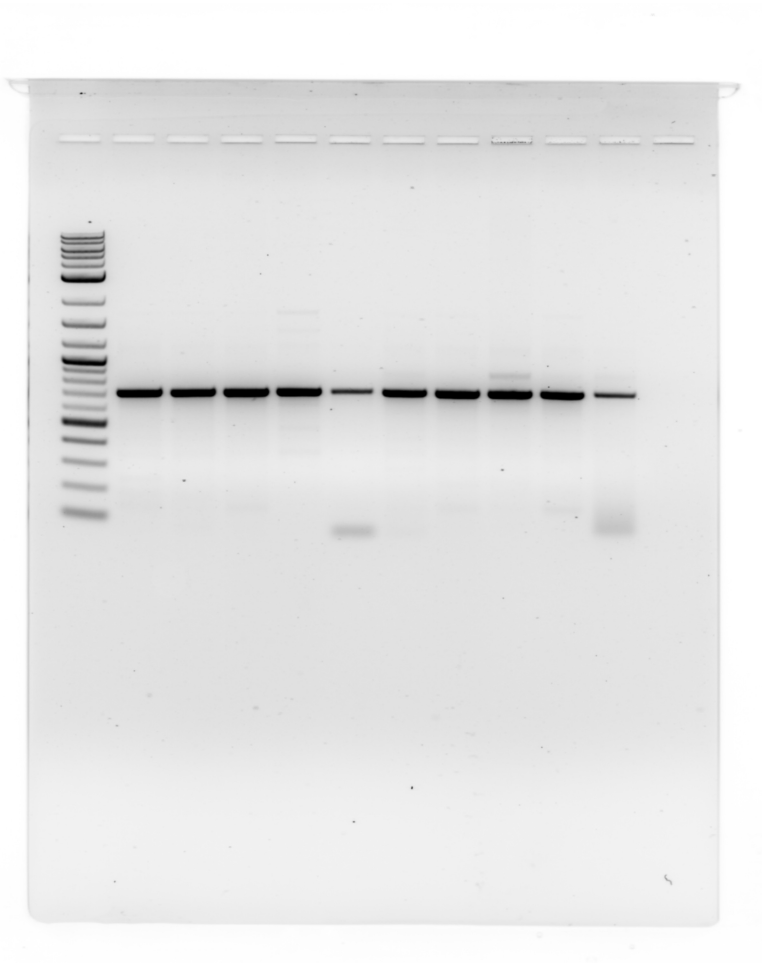

Figure S1C – anti-proteasome 20S alpha subunits 1,2,3,5,6, and 7 (Enzo Life Sciences BML-PW8195-0100). All lanes in figure.

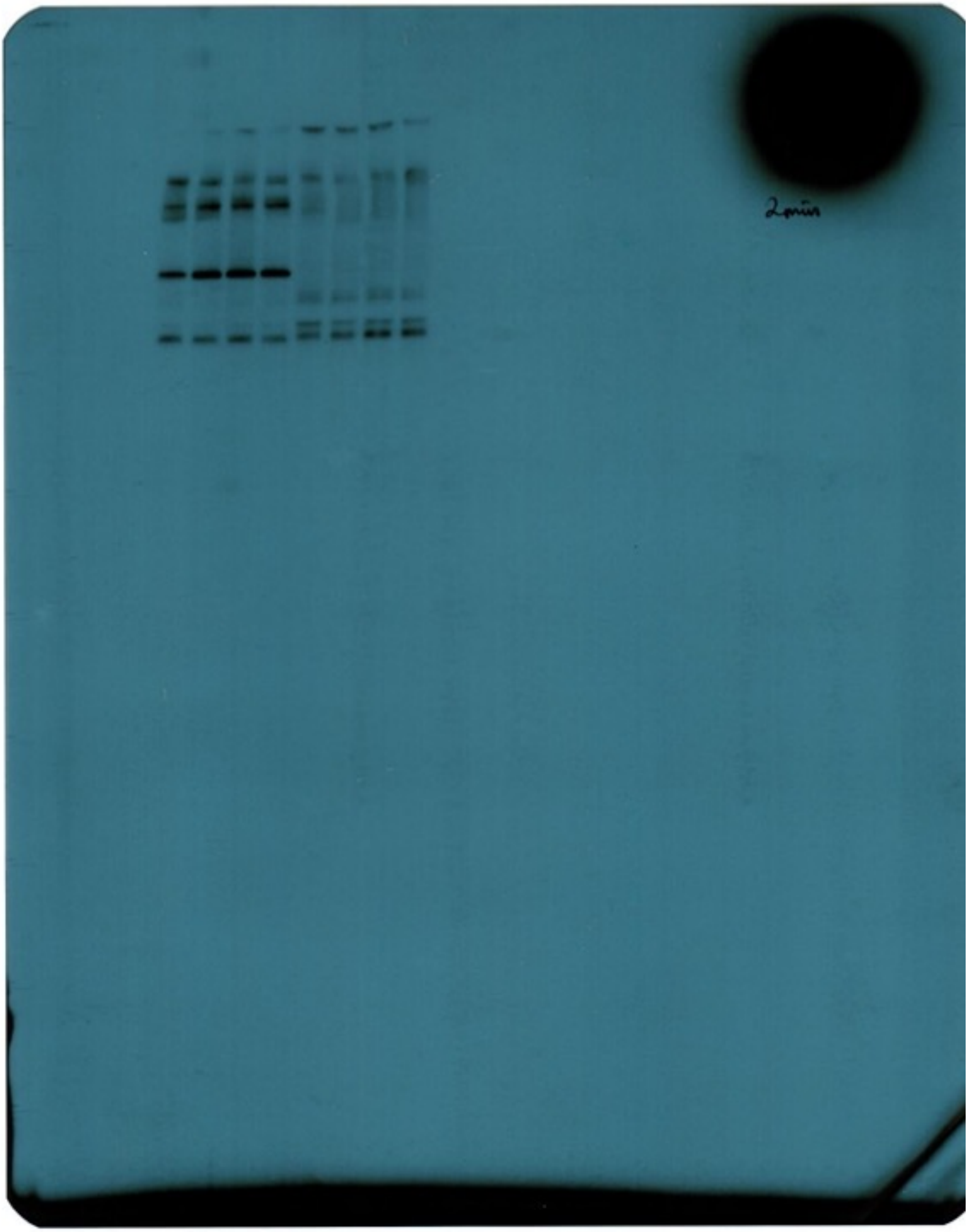

Figure S1D – Ponceau S staining for blot in S1C, all lanes in figure

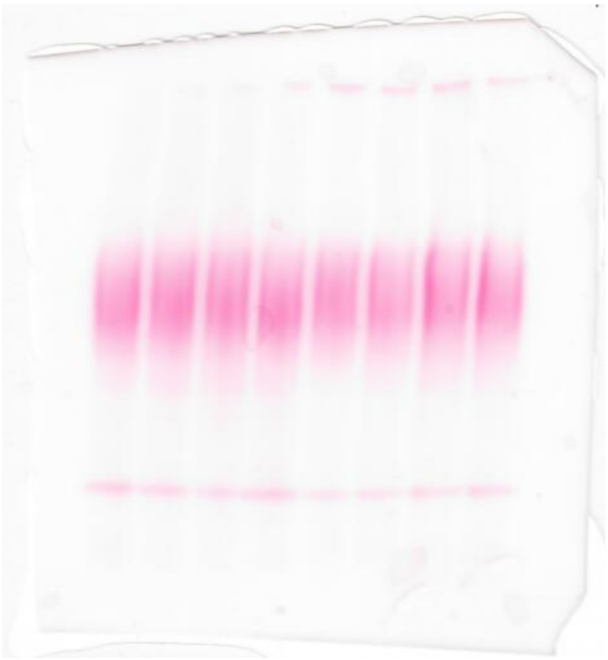

Supplement: Unedited blot and gel images [file jciinsight-9-181723-s138.pdf]
